# The Majority of MicroRNAs Detectable in Serum and Saliva Is Concentrated in Exosomes

**DOI:** 10.1371/journal.pone.0030679

**Published:** 2012-03-09

**Authors:** Alessia Gallo, Mayank Tandon, Ilias Alevizos, Gabor G. Illei

**Affiliations:** Sjögren's Syndrome Clinic, Molecular Physiology and Therapeutics Branch, National Institute of Dental and Craniofacial Research, National Institutes of Health, Bethesda, Maryland, United States of America; University of Bradford, United Kingdom

## Abstract

There is an increasing interest in using microRNAs (miRNA) as biomarkers in autoimmune diseases. They are easily accessible in many body fluids but it is controversial if they are circulating freely or are encapsulated in microvesicles, particularly exosomes. We investigated if the majority of miRNas in serum and saliva are free-circulating or concentrated in exosomes. Exosomes were isolated by ultracentrifugation from fresh and frozen human serum and saliva. The amount of selected miRNAs extracted from the exosomal pellet and the exosome-depleted serum and saliva was compared by quantitative RT-PCR. Some miRNAs tested are ubiquitously expressed, others were previously reported as biomarkers. We included miRNAs previously reported to be free circulating and some thought to be exosome specific. The purity of exosome fraction was confirmed by electronmicroscopy and western blot. The concentration of miRNAs was consistently higher in the exosome pellet compared to the exosome-depleted supernatant. We obtained the same results using an equal volume or equal amount of total RNA as input of the RT-qPCR. The concentration of miRNA in whole, unfractionated serum, was between the exosomal pellet and the exosome-depleted supernatant. Selected miRNAs, which were detectable in exosomes, were undetectable in whole serum and the exosome-depleted supernantant. Exosome isolation improves the sensitivity of miRNA amplification from human biologic fluids. Exosomal miRNA should be the starting point for early biomarker studies to reduce the probability of false negative results involving low abundance miRNAs that may be missed by using unfractionated serum or saliva.

## Introduction

Many of the systemic autoimmune diseases have heterogeneous clinical presentations making accurate diagnosis and monitoring of clinical activity difficult. Therefore, there is a need to identify and validate non-invasive biomarkers, which can be used to improve the accuracy of diagnosis, predict prognosis and to monitor disease progression and response to therapy.

MicroRNAs (miRNAs) are small regulatory non-coding RNAs with important roles in a variety of physiological and pathological processes. Among others they are instrumental in regulating immune development, normal immune function and autoimmunity. MiRNAs can be readily isolated from fresh or fixed tissues and body fluids. Their expression patterns reflect the pathophysiological status of a tissue [1] and have been shown to be specific for particular disease states. Additionally, miRNAs are more stable than mRNAs and thus less prone to minor differences in sample processing. Together these characteristics make them excellent biomarker candidates. Easily accessible body fluids such as blood derivatives and saliva or urine would provide an ideal source for miRNA biomarkers. It was shown previously that miRNA signatures from plasma, serum and whole blood were not significantly different [2], [3] and it was hypothesized that miRNAs were encapsulated in separate structures.

Exosomes are small microvesicles, about 30–100 nm in size [4]. They are secreted by a variety of cell types such as epithelial cells, B- and T-lymphocytes [5], mast cells [6], dendritic cells [6], and neurons [7] and carry proteins and nucleic acids. MiRNA signatures from both unfractionated whole serum, urine, saliva, cerebrospinal fluid [8] and from exosomes [9] showed promise as diagnostic biomarkers, but there is no consensus about the relative contribution of exosomal miRNAs to whole serum microRNAs. Determining this would have important practical implications on miRNA biomarker studies as well as studies exploring the biologic function of circulating miRNAs.

The goal of this study was to determine if miRNAs found in serum and saliva are primarily in exosomes and whether there is any benefit of using exosomes over unfractionated biologic fluids in biomarker studies. In contrast to two recent papers [10], [11] which claimed that the majority of miRNAs found in plasma and serum is present primarily outside exosomes here we demonstrate that miRNAs in serum and saliva exist primarily inside exosomes and that using exosomal fraction increases the sensitivity of miRNA detection.

## Results

### Exosome isolation

**Figure 1:**
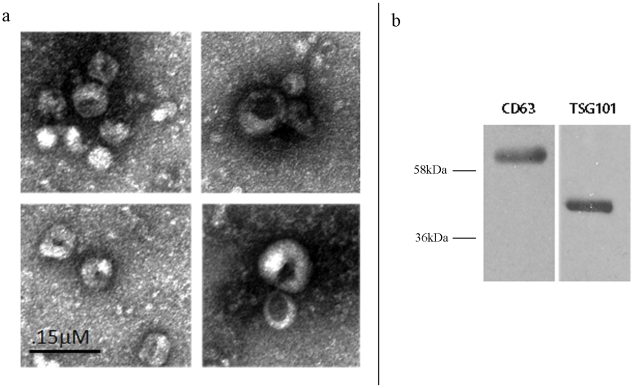
Confirmation that the ultracentrifugation pellet contains exosomes. **a** Electron microscopy of the ultracentrifugation pellet from serum shows the characteristic spherical shape and size (50–100 nm) of exosomes b. Western blot shows strong staining of the ultracentrifugation pellet with the exosomal membrane markers anti cd63 and TSG101.

We first optimized an ultracentrifugation protocol to isolate exosomes from small amounts (<1 mL) of fresh and frozen human serum and saliva. Electron microscopic analysis of the pellet showed spherical structures with a size varying between 50–110 nm (Fig. 1a), consistent with previously reported characteristics of exosomes [12]. We further confirmed that these vesicles are exosomes by performing Western blot analysis on lysates of the ultracentrifugation pellets using antibodies against two commonly used exosomal markers, the tetraspanin molecule CD63, and TSG101 [13], [14] (Fig. 1b).

### Majority of miRNAs are within exosomes in human serum and human saliva samples

To determine if the miRNA in serum or saliva is contained in exosomes or is circulating freely, we extracted the RNA from the exosomes in the pellet and from the exosome-depleted supernatant from both serum and saliva. The optimized exosome isolation method allowed us to start from small volumes of samples (300 µl - 1 ml) and to use the whole exosomal pellet and the entire volume of the supernatant for RNA isolation. The RNA isolated from both sources was dissolved in a volume of 25 µL. The amount of selected miRNAs was compared by determining their threshold cycle (Ct) by real-time quantitative (RT-qPCR). The Ct is defined as the cycle number at which the fluorescence emission exceeds a fixed threshold. A Ct of 35 was considered the lower level of detection. Undetectable miRNAs were given a Ct value of 40. The miRNAs tested in serum and saliva samples are shown in Table 1. These miRNAs were selected because they are either ubiquitously expressed or have been reported as biomarkers (Table 1). The relative concentration of these microRNAs was determined by calculating the difference of Ct values between the exosome samples and the exosome-depleted supernatant. A 1-unit difference in the Ct value between the miRNAs isolated from the exosomal pellet or supernatant represents a 2-fold difference in the amount of input miRNA.

**Table 1:** List of miRNAs tested in serum and saliva samples.

Serum miRNA	Previously associated with or described as	Saliva miRNA	Previously associated with or described as
let-7a	Exosomal miRNA ^(11)^	miR-22	Ubiquitous
miR-92a	Free circulating miRNA ^(11)^	miR-202	Ubiquitous
miR142-3p	Exosomal miRNA ^(11)^	miR-203	Colorectal cancer
miR-101	Gastric cancer- lung cancer	miR-1273d	Present in salivary glands
miR-16	CLL		
miR-107	Alzheimer		
miR-122	Viral hepatitis		
miR-574	Mononuclear infiltrates in SS		
miR-768	Mononuclear infiltrates in SS		

**Figure 2:**
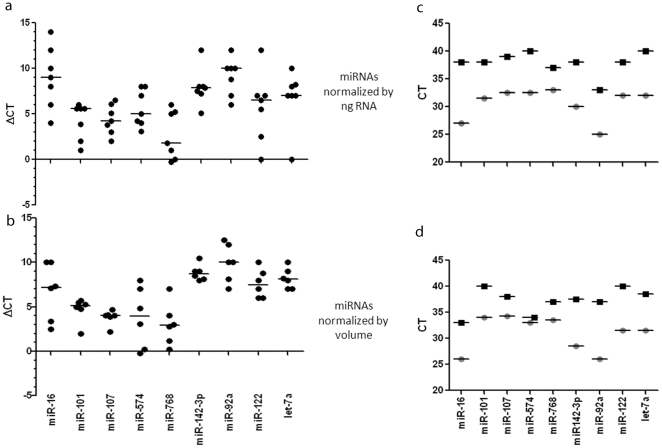
Serum miRNAs are predominantly in exosomes. The relative expression of microRNAs extracted from exosome-depleted supernatant or exosomes was expressed as the difference in threshold cycle number between the exosome depleted supernatant and the exosome pellet (ΔCt: Ct supernatant – Ct exosome pellet). A 1 unit difference in ΔCT represents a two-fold difference in the amount of input miR. Positive numbers show higher concentrations in the exosomes whereas negative numbers indicate higher concentrations in the exosome-depleted supernatant. The absolute difference between the two can be calculated as 2^ΔCT^. The RNA included in the qPCR reaction was normalized to either equal amount (**a**) or equal volume (**b**) of RNA. Panels (**c**) and (**d**) show the absolute differences in microRNA levels amplified from serum exosome pellet (○) and exosome-depleted supernatant (□).

To explore the effect of various normalization approaches we performed two sets of RT-qPCRs on miRNAs derived from sera normalizing for input RNA or original volume. First we retrotranscribed 10 ng of RNA for each sample. Using the same amount of RNA avoids artifacts that may result from including different amounts in the qRT-PCR reaction. This is particularly important for RNAs present at low copy numbers. However, this normalization may lead to a disproportionate representation of the original volumes these RNAs were derived from. In fact, since the concentration of RNA derived from the supernatant was higher than the exosomal RNA, equal amounts of exosomal RNA represents a larger volume of serum than the RNA form the supernatant. To address this, we repeated these experiments using the same volume of RNA, which represents the same volume of serum for both the pellet and the supernatant. As shown in Figure 2a, the concentration of the miRNAs was consistently higher in the exosomes regardless of the normalization. The mean in the pellet is at least 6 fold (2.2 cycles) higher than in the supernatant for all the miRNAs tested. The difference ranges from 6–128 folds (2.2–12.5 cycles). To put it differently, 83% to more than 99% of miRNAs is detected in the exosomal fraction. In fact many miRNAs were under the lower range of reliable detection (Ct>35) in the exosome-depleted supernatant. We obtained similar results when we used the same volume rather than the same amount of miRNA for the RT-qPCR (Figure 2b). Together these data show that the majority of miRNAs are contained in exosomes and there is no substantial difference whether we use equal amounts or volume of input RNA. The rest of the data presented in this paper show the results of experiments using equal amounts of RNA.

Figure 2c and 2d show the average of threshold cycles for each miRNA obtained using the same amount of RNA (2c) or the same volume of sample (2d). The Ct value for the majority of miRNAs was above 35 cycles, which is considered as the threshold for reliable detection of miRNAs, showing that most miRNAs tested are either absent or present at a very low level outside exosomes but can be reliably detected in isolated exosomes.

**Figure 3:**
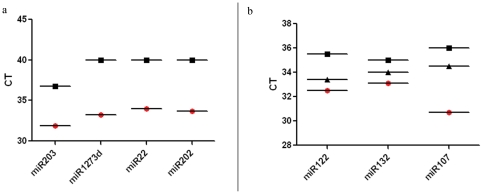
Salivary miRNAs are predominantly in exosomes. (**a**) Absolute differences in microRNA levels amplified from saliva exosome pellet (○) and exosome-depleted supernatant (□). 1 unit increase in CT represent a two-fold lower amount of miRNA. miRNAs requiring 35 cycles or more are considered undetectable. (**b**) Graph showing the average of three samples in microRNA levels amplified from serum exosome pellet (○) and exosome-depleted supernatant (□) and whole serum (▵). Ct value for the whole serum samples was consistently lower (by at least 2 cycles) than the supernatant and at least 1.5 cycles higher than the exosome pellet samples.

To evaluate if this observation is applicable to other biologic fluids we also tested miRNA concentration in salivary exosomes and exosome-depleted supernatants of saliva. The miRNAs tested in the saliva samples (Table 1) were previously shown to be present in saliva or salivary glands (Fig. 3a). Overall, the concentration of these miRNAs was lower in salivary exosomes compared to the ones tested in the serum. In the saliva samples, 3/4 of the miRNAs tested were undetectable (Ct>40 cycles). The one miRNA that was detectable in the exosome-depleted supernatant was also above the recommended threshold for detection and was 32-fold (5 cycles) more concentrated in exosomes.

### MiRNA in whole serum

Next we addressed the question if there was a potential benefit in using exosomal miRNA over whole serum to detect circulating miRNAs. To answer this question, we processed half the samples as previously described (exosome isolation and RNA extraction) whereas from the other half we extracted RNA directly, skipping the exosome isolation step (whole serum).

As shown in Figure 3b the Ct value for the whole serum samples was consistently lower (by at least 2 cycles) than the supernatant and at least 1.5 cycles higher than the exosome pellet samples.

## Discussion

Together these data demonstrate that the exosome fraction of serum and saliva is highly enriched in miRNAs, and that the majority of this class of RNA is not freely circulating in human biologic fluids but is enclosed in microvesicles, primarily exosomes. The finding that miRNAs are primarily in exosomes was consistent for all miRNAs tested, for both fresh and frozen serum (data not shown), for various biologic fluids (serum and saliva) and across all healthy individuals tested and the patient with systemic lupus erythematosus. This finding is in apparent contrast with two recent publications, which found that miRNAs are predominantly exosome-free in cell culture media [10], and bound to Argonaut-2 protein in plasma and serum [11]. The reason for this difference is unclear but may, at least in part, be due to minor differences in isolating exosomes or differences between plasma and serum. In fact Arroyo et al showed that in contrast to plasma, in serum miRNA was more commonly found in exosomes than circulating freely. To address this discrepancy we tested some of the miRNAs, which were studied by Arroyo et al (let-7a, miR-92a, miR142-3p). In contrast to their study, which found that some are primarily in exosomes whereas others are circulating freely, we found that the all of these miRNAs are primarily in exosomes. The reason for this difference may be due to low grade lysis of exosomes during the exosome isolation process.

Comparing miRNA detection from whole serum and isolated exosomes showed that exosome isolation improves the sensitivity of miRNA amplification from human biologic fluids. This difference was relatively small for some miRNAs but for one (miR107) using exosomal miRNA substantially increased the sensitivity of detection. We believe that the extra effort required for exosomes isolation is justified for many applications. Exosomal miRNA should be the starting point for early biomarker studies to reduce the probability of false negative results involving low abundance miRNAs that may be missed by using unfractionated serum or saliva. Once potential biomarkers are identified in exosomes, methods can be optimized to detect these specific miRNAs in whole serum or saliva for use in larger studies and everyday practice.

## Materials and Methods

### Blood samples

The study was approved by the Institutional Review Boards of the National Institute of Diabetes Digestive and Kidney Diseases and the National Institutes of Arthritis and Musculoskeletal and Skin Diseases and the National Institute of Dental and Craniofacial Research. All subjects have signed a written informed consent. Serum was isolated from 6 healthy volunteers and 1 patient with systemic lupus erythematosus. Briefly, 10 ml of blood was collected in a serum separator tube and processed within an hour. Separation of the serum was accomplished by centrifugation at 800× g for 10 minutes at room temperature. The serum obtained was further processed for exosome isolation. Saliva samples: saliva was collected in a sterile tube and directly processed for the exosome isolation as described in the following paragraph.

### Exosome isolation

Fluid samples were centrifuged at 1500× g for 10 minutes. The collected supernatant was centrifuged again at 17,000× g for 15 minutes, and the supernatant was spun again in an ultracentrifuge at 160,000× g for 1 hour. All centrifugations were done at 4°C. The pellet containing exosomes and the exosome-depleted supernatant were then processed to extract RNA.

### RNA Extraction and Reverse Transcription

RNA was extracted from the pellet and from the exosome-depleted supernatant using the TRIZOL reagent (Invitrogen, Carlsbad, CA) according to the protocol provided by the manufacturer. Briefly, 1.0 ml TRIZOL reagent and 200 µl chloroform were added to the sample and the mixture was vortexed for 15 s and stood at 25°C for 3 minutes. After centrifugation at 12000× g for 15 minutes at 4°C, the supernatant was transferred to a fresh tube and 500 µl isopropanol was added. After incubation at −20°C for 20 minutes, the mixture was centrifuged at 12000× g for 10 minutes at 4°C to remove the supernatant and the RNA pellet was washed with 75% ethanol. After removal of ethanol by centrifugation at 7500× g for 5 minutes at 4°C, RNA was air-dried for 5 minutes and then dissolved in 25 µl RNase-free water. The purity of isolated RNA was determined by OD260/280 using a Nanodrop ND-1000 (Thermo Scientific, Worcester, MA). Each RNA sample was polyadenylated and reversely transcribed to cDNA using the TaqMan® MicroRNA Reverse Transcription Kit (Applied Biosystems, Foster City, CA). Single-stranded cDNA was synthesized from 10 ng of total RNA (normalization by amount) or from 1 µl of the RNA volume (normalization by volume) using specific miRNA primers (TaqMan MicroRNA Assay, PN 4427975, Applied Biosystems).

### miRNA quantification

Two µl of cDNA was used as a template in a 15 µl PCR reaction. PCR products were amplified using specific primers (TaqMan MicroRNA Assay) and the TaqMan Universal PCR Master Mix (PN 4324018, Applied Biosystems), and detected using StepONE plus Real time PCR system (Applied Biosystems). PCR reactions for each sample were run in triplicate, including blank controls without cDNA. The following TaqMan MicroRNA Assays used in this study were obtained from Applied Biosystems and used miRNA detection: hsa-let-7a (000377), hsa-miR-92a (000431), hsa-miR-142-3p (000464) miR101 (000438), miR107 (000443), miR122 (000445), miR574 (002349), miR16 (000391), miR678 (000449) for serum and miR203 (000507), miR22 (000398), miR202 (001012), and miR1273d (000437) for saliva.

### Western blot immunoblotting

Proteins were extracted from the ultracentrifugation pellets and separated on a polyacrylamide gel before transfer to a PVDF membrane. The blotting membrane was blocked with bovine serum albumin and incubated with CD63 antibody (Abcam, Cambridge, UK) and with TSG101 (Abcam, Cambridge UK) followed by incubation with horseradish peroxidase-coupled secondary antibody (Cell Signaling Technology, Inc. Danvers, MA). The proteins were detected using enhanced chemiluminescence (Thermo Scientific, Rockford, USA).

## References

[1] Alevizos I, Illei GG (2010). MicroRNAs as biomarkers in rheumatic diseases.. Nat Rev Rheumatol.

[2] Kroh EM, Parkin RK, Mitchell PS, Tewari M (2010). Analysis of circulating microRNA biomarkers in plasma and serum using quantitative reverse transcription-PCR (qRT-PCR).. Methods.

[3] Mitchell PS, Parkin RK, Kroh EM, Fritz BR, Wyman SK (2008). Circulating microRNAs as stable blood-based markers for cancer detection.. Proc Natl Acad Sci U S A.

[4] Heijnen HF, Schiel AE, Fijnheer R, Geuze HJ, Sixma JJ (1999). Activated platelets release two types of membrane vesicles: microvesicles by surface shedding and exosomes derived from exocytosis of multivesicular bodies and alpha-granules.. Blood.

[5] McLellan AD (2009). Exosome release by primary B cells.. Crit Rev Immunol.

[6] Admyre C, Telemo E, Almqvist N, Lötvall J, Lahesmaa R (2008). Exosomes - nanovesicles with possible roles in allergic inflammation.. Allergy.

[7] Lachenal G, Pernet-Gallay K, Chivet M, Hemming FJ, Belly A (2011). Release of exosomes from differentiated neurons and its regulation by synaptic glutamatergic activity.. Mol Cell Neurosci.

[8] Tzimagiorgis G, Michailidou EZ, Kritis A, Markopoulos AK, Kouidou S (2011). Recovering circulating extracellular or cell-free RNA from bodily fluids.. Cancer Epidemiol.

[9] Michael A, Bajracharya SD, Yuen PS, Zhou H, Star RA (2010). Exosomes from human saliva as a source of microRNA biomarkers.. Oral Dis.

[10] Turchinovich A, Weiz L, Langheinz A, Burwinkel B (2011). Characterization of extracellular circulating microRNA.. Nucleic Acids Res.

[11] Arroyo JD, Chevillet JR, Kroh EM, Ruf IK, Pritchard CC (2011). Argonaute2 complexes carry a population of circulating microRNAs independent of vesicles in human plasma.. Proc Natl Acad Sci U S A.

[12] Silverman JM, Reiner NE (2011). Exosomes and other microvesicles in infection biology: organelles with unanticipated phenotypes.. Cell Microbiol.

[13] Théry C, Zitvogel L, Amigorena S (2002). Exosomes: composition, biogenesis and function.. Nat Rev Immunol.

[14] Lakkaraju A, Rodriguez-Boulan E (2008). Itinerant exosomes: emerging roles in cell and tissue polarity.. Trends Cell Biol.

